# Circadian Rhythm Neuropeptides in *Drosophila*: Signals for Normal Circadian Function and Circadian Neurodegenerative Disease

**DOI:** 10.3390/ijms18040886

**Published:** 2017-04-21

**Authors:** Qiankun He, Binbin Wu, Jeffrey L. Price, Zhangwu Zhao

**Affiliations:** 1 Department of Entomology, China Agricultural University, 2# Yuanmingyuan West Road, Beijing 100193, China; qiankunhe@cau.edu.cn (Q.H.); binbinwu@cau.edu.cn (B.W.); 2 Division of Molecular Biology and Biochemistry, School of Biological Sciences, University of Missouri–Kansas City, 5100 Rockhill Rd., Kansas City, MO 64110, USA

**Keywords:** circadian rhythm mechanism, circadian neuropeptides, PDF, NPF, ILP, Alzheimer’s disease, tauopathy, apoptosis

## Abstract

Circadian rhythm is a ubiquitous phenomenon in many organisms ranging from prokaryotes to eukaryotes. During more than four decades, the intrinsic and exogenous regulations of circadian rhythm have been studied. This review summarizes the core endogenous oscillation in *Drosophila* and then focuses on the neuropeptides, neurotransmitters and hormones that mediate its outputs and integration in *Drosophila* and the links between several of these (pigment dispersing factor (PDF) and insulin-like peptides) and neurodegenerative disease. These signaling molecules convey important network connectivity and signaling information for normal circadian function, but PDF and insulin-like peptides can also convey signals that lead to apoptosis, enhanced neurodegeneration and cognitive decline in flies carrying circadian mutations or in a senescent state.

## 1. Introduction

An endogenous rhythm is an autonomous rhythm, which is usually studied in a constant environmental condition, thereby demonstrating that it arises from cycling organismal processes rather than from cycling environmental conditions. The word “circadian” stems from the Latin term circa diem, which means “for about a day” [1]. Thus, the term circadian rhythm can be interpreted as a biological cycle (e.g., a daily activity rhythm) that is set by an internal timing mechanism. Despite their endogenous nature, the phase of a circadian rhythm is set by the oscillation of external environmental factors, like light, temperature and food; the phase-setting by these environmental factors is termed “entrainment” and is analogous to resetting a fast or slow clock every day by a manual readjustment of the hands.

In 1971, three mutants (*per^0^*, *per^s^* and *per^l^*), in which the free-running period of the circadian rhythm was either shortened or prolonged and for one of them was arrhythmic [2], were isolated. Since then, a series of clock-related genes was identified and characterized [3,4,5,6,7,8,9,10,11,12,13,14,15,16]. Subsequently, a large number of studies showed that these clock genes and proteins regulate the *Drosophila* circadian rhythm in a genetic pathway and established the classical transcriptional feedback loops that are widely accepted as keeping circadian time in animals. These core clock loops have been discussed in a number of reviews [17,18,19,20,21] and therefore are only briefly summarized here.

**Figure 1:**
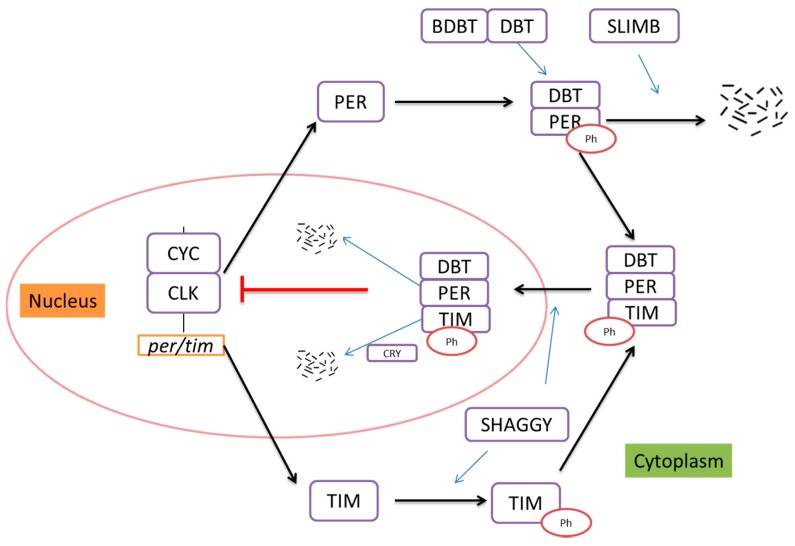
Model of circadian clock in *Drosophila*. CLK and CYC form a heterodimer promoting the transcriptions of *per* and *tim* mRNA. PER and TIM accumulate in the cytoplasm and associate with each other and DBT to constitute the PER/TIM/DBT complex. This complex enters the nucleus to suppress the CLK/CYC heterodimer. When TIM is degraded by CRY in response to light, DBT promotes the degradation of nuclear PER, thereby de-repressing CLK/CYC function and starting a new cycle of transcription. BDBT, DBT and SLIMB adjust the accumulation of PER in the cytoplasm, and SHAGGY phosphorylates TIM to promote nuclear entry of the PER/TIM/DBT complex. This figure summarizes the discussions of [17,18,19,20,21]. Arrows represent positive action, and T-bars represent negative action. CLK, clock; CYC, cycle; PER, period; TIM, timeless; DBT, doubletime; BDBT, bride of doubletime; CRY, cryptochrome.

These feedback loops are both positive and negative, with positive factors (e.g., clock (CLK) and cycle (CYC)) producing transcription of negative factors (e.g., period (PER) and timeless (TIM)). The transcription factors CLK and CYC form a heterodimer that binds to the E-box in the *per* and *tim* promoters, thereby promoting transcription of these genes [9,22]. Cytoplasmic PER protein is phosphorylated by doubletime (DBT) and as a consequence becomes vulnerable to degradation [6] due to the effects of the Slimb E3 ligase [23]. Bride of doubletime (BDBT) binds to DBT to stimulate its activity towards PER [7], but TIM protein, which accumulates during the night, can bind with PER to constitute the PER/TIM/DBT complex, thereby preventing DBT-dependent PER degradation and stabilizing PER [24]. With the help of Shaggy [11], the complex enters the nucleus in the second half of the night to suppress the function of the CLK/CYC heterodimer [25]. TIM is degraded in response to light due to a light-dependent interaction with an intracellular flavoprotein photoreceptor call cryptochrome (CRY). After TIM is degraded in the early morning by CRY in response to light [10], DBT promotes the degradation of nuclear PER, and the CLK/CYC function is recovered, starting a new round of expression of *per*, *tim* and a large number of CLK/CYC-driven genes whose oscillations confer the outputs of the circadian clock (Figure 1). PER and TIM proteins oscillate in level out of phase with their respective RNAs, which peak in the early evening while nuclear PER and TIM proteins peak in late night and early morning. This unusual phase relationship is thought to be produced at least in part by the coupling of the transcriptional negative feedback loop with the post-translational effects of CRY, phosphatase and kinases like DBT, which delay that initial accumulation of PER and TIM until the *per* and *tim* mRNAs have reached their peak levels of expression. Subsequent transcriptional repression is produced by the delayed nuclear accumulation of PER and TIM, thereby producing the oscillations [6].

In addition, there are several genes constituting interlocked loops with core clock genes (for details, see the review [18]), such as *par domain protein 1* (*pdp1*) [3], *vrille* (*vri*) [13], *E75* [26] and *clockwork orange* (*cwo*) [27,28]; these are thought to contribute to the robustness and fine-tuning of the core molecular clock feedback loops. In addition to the above elements, post-transcriptional and translational control mechanisms have been shown to contribute to the expression of circadian genes [29,30,31]. These mechanisms include regulation of mRNA stability, mRNA splicing and *dbt* and *per* translation.

Here, we focus on the analysis of circadian rhythms in *Drosophila melanogaster*, review how the circadian feedback loops regulate or are regulated by neurotransmitter and neuroendocrine signals and discuss the interrelation between the circadian clock, circadian neuropeptides and neurodegenerative disease.

## 2. The *Drosophila* Circadian Rhythm and Neuroendocrine Regulation

The circadian system synchronizes metabolic, physiological and behavioral functions by organizing a temporal segregation, which avoids the simultaneous occurrence of conflicting behaviors or conflicting cellular functions [32]. Circadian regulation of physiology and behavior therefore results from the coordination of the activities of multiple tissues and cell types [17]. In the fly brain, circadian cells consist of approximately 150 clock neurons. These neurons include three groups of dorsal neurons (DN1, DN2, DN3), one group of dorsal lateral neurons (LNd), two groups of ventral lateral neurons (LNv) and three lateral posterior neurons on both sides of the brain. The LNvs are divide into four large ventral lateral neurons (l-LNv), four small ventral lateral neurons (s-LNv) (both types of which express the pigment dispersing factor (PDF)) and a fifth small LNv that does not express PDF. Previous studies demonstrated that a network containing the LNv neurons coordinates morning activity, while a network containing other neurons (the PDF, fifth LNv, the LNds and two DN1s) coordinates evening activity [33,34]. There is no doubt these networks need outputs for their communication, and neuropeptides and neurotransmitters as key mediators of many behavioral and physiological processes may play this role. These output molecules that influence or are influenced by circadian rhythms include peptides, hormones and biogenic amines [35].

The most extensively studied ‘‘circadian neuropeptide’’ is pigment dispersing factor (PDF), which has a complex relationship with the clock system [36,37,38,39,40,41,42]. On the one hand, PDF is an output factor of the large and small LNv [43] and exhibits strong regulation of sleep and awake in *Drosophila* [42,44,45]. *Pdf* mRNA levels are cycling except under light-dark (LD) conditions, and PDF accumulation and release seem to be regulated by clock systems in a rhythmic manner [36]. On the other hand, PDF feeds back on the circadian clock system in clock cells that express its receptor [46]. From the large number of studies on PDF, we can give this summary: PDF-expressing neurons receive the environmental or circadian signal and transduce this signal to the release of PDF to PDF receptor-expressing neurons, which then change their cAMP levels internally through the actions of their G-protein-coupled receptors (GPCR), bringing about an effect on the circadian molecular oscillations or period through changes in PER/TIM levels [47].

Of course, the relation between PDF and the clock system is not as simple as just described. There are many additional neuropeptides that have been identified to play a role in the circadian clock system of *Drosophila*, such as neuropeptide F (NPF), short neuropeptide F (sNPF) and ion transport peptide (ITP) [48,49]. Our laboratory has done detailed research on NPF’s role in the *Drosophila* circadian rhythm. We showed that NPF is expressed in dorsal neurons (DNs) including DN1 and DN2, dorsal-lateral neurons (LNds), ventral-lateral neurons (LNvs) including large l-LNvs and small s-LNvs and other non-clock neurons, while its G protein-coupled receptor NPFR1 is located in DN1 and LNds. We further monitored locomotor activity in LD cycles and under continuous constant dark (DD) conditions in transgenic flies with NPF and NPFR1 downregulation, and we found that anticipatory behavior in the evening disappeared, a phenotype consistent with the functions of LNds and the fifth s-LNv on evening activity [50]. Thus, we concluded that NPFR1 mediates NPF participation in the regulation of circadian rhythm [48]. It is worth noting that one of the NPF-positive LNds also expresses CRY and ITP, and some peripherally rhythmic genes, like the cytochrome P450 family and sex-specific enzyme 1 (sxe1), were reported to be under the control of NPF signaling [49,51,52].

As for sNPF, we [53] and another group [54] demonstrated that sNPF regulates *Drosophila* sleep. We showed that it does so through a cAMP-PKA-CREB pathway. Moreover, sNPF and sNPFR are distributed in the mushroom body (MB), pars intercerebralis (PI) neurons [53] and a subset of clock neurons, including PDF-positive s-LNvs and two pairs of NPF-negative LNds [49], which imply their potential functions in circadian rhythm. One recent paper also showed that the two sNPF- and PDF receptor (PDFR)-positive LNd neurons per hemisphere, rather than other PDFR LNds or the fifth s-LNv, were coupled to PDF neurons and triggered coupling of the morning oscillators [55].

*Drosophila* Insulin-like peptides (DILPs) may also play a role in sleep-awake cycles, since DILP-2 was detected in l-LNvs and s-LNvs, and phenotypes associated with sleep regulation were reported [56]. *Drosophila* insulin-producing cells (IPCs) are also located in the PI and connected to the central circadian clock circuit via DN1 neurons [57]. Reduction of the insulin pathway rescues circadian and memory defects in the fragile X mutant fly [58]. It has been reported that insulin mediates metabolic clock output through regulating the rhythmic expression of a metabolic gene (sxe2) in the fat body [57].

The ion transport peptide (ITP) is expressed in one of the LNds and the fifth LNv that is PDF negative. Altered levels affect the phase of the evening activity peak, suggesting that it may be the neuropeptide that regulates the activity of the evening oscillator, while PDF regulates the activity of the morning oscillator [49,59].

Other neuropeptides, like diuretic hormone 31 (DH31), diuretic hormone 44 (DH44), leucokinin neuropeptide (LK) and allatostatin A (AstA), are not found in the clock neurons, but also contribute to circadian rhythms. Although DH31 homozygous mutants still exhibit robust free-running activity rhythms and an ~24-h period [60], the effects on a temperature preference rhythm through PDFR in DN2s were demonstrated last year [61] and also suppression of sleep late at night [60]. Lateral horn leucokinin neuropeptide (LHLK), leucokinin neuropeptide receptor (LK-R) and DH44 display clock-dependent activity rhythms in explanted brains on the downstream clock network [62], and DH44 has been shown to signal rhythmic locomotor activity from the PI in response to circadian pacemaker cell inputs to the PI [63]. The AstA neurons regulating feeding and sleep in *Drosophila* are verified as down-stream targets of PDF [64].

One other thing to note is that the function of the neuropeptides is convergent. Most endocrines act on GPCRs, important regulators of intracellular levels of cAMP, which has an essential role in intracellular signaling [65,66]. It has been established that cAMP levels are central to circadian clock function [39,40,67,68,69], thereby firmly supporting the relationship between neuropeptides and the circadian clock.

**Figure 2:**
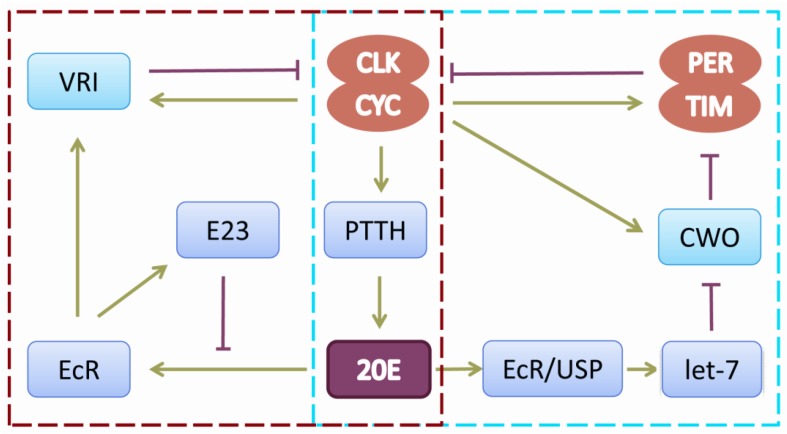
Ecdysone-mediated signaling contributes to circadian regulation. CLK/CYC directly controls the expression of *ptth*, and ecdysone hormone is subsequently stimulated to act on miRNA let-7 through EcR/USP in circadian neurons. miRNA let-7 affects the locomotors rhythm by suppressing the circadian-relevant target gene cwo, which encodes a transcriptional repressor competing with CLK/CYC binding to E-box sequences. On the other hand, ecdysone and E23 form a feedback loop mediating the level of VRI, which binds on the promoter region of *clk* as a repressor. Arrows represent positive action, and T-bars represent negative action. Two different colors of dotted boxes represent two pathways, respectively. CLK, clock; CYC, cycle PTTH, prothoracicotropic hormone; 20E, ecdysone hormone; EcR, ecdysone hormone receptor; USP, ultraspiracle protein; CWO, clockwork orange; E23, early gene at 23; VRI: vrille. PER, period; TIM, timeless.

Ecdysone is amongst the best studied hormones in *Drosophila* and is well characterized with respect to its essential role in coordinating developmental transitions such as larval molting and metamorphosis [70]. Ecdysteroid biosynthesis and signaling are also active in adult insects, and we have reported that CLK-PTTH-ecdysone-ecdysone receptor (EcR)/ultraspiracle protein (USP)-mediated signaling contributes to circadian regulation [71]. CLK/CYC has a direct transcriptional effect on the expression of prothoracicotropic hormone (*ptth*), and the *ptth* transcriptional periodicity is correlated with ecdysteroid titer [72]. EcR/USP in circadian neurons receives ecdysone and binds to the endogenous let-7-C locus, thereby activating its transcription, and the miRNA let-7 affects the locomotor rhythm by suppressing the circadian-relevant target gene *cwo* in the central clock regulatory cycle. In addition, VRI also responds to ecdysone both in cell culture and in vivo [73]. Early gene at 23 (E23) and ecdysone form a feedback loop to tune a circadian oscillation, in which *E23* is induced by ecdysone hormone, and its protein negatively regulates ecdysone-mediated *vri* expression [74]. These investigations highlight the role of ecdysone on circadian regulation (Figure 2). The circadian clock is a key driver of steroid hormone production in *Drosophila* [75]. Other hormones also have similar roles in the circadian system, such as juvenile hormones (JHs) and melatonin (*N*-acetyl-5-methoxytryp-tamine) (for the details, see the review [35]).

Biogenic amines, including octopamine, serotonin and dopamine, are expressed or function in different clock neurons in the brain of *Drosophila* and influence or are influenced by the circadian clock as neurotransmitters [76,77,78]. Serotonin acting through the serotonin receptor 1B modulates circadian entrainment. This serotonin receptor is expressed in clock neurons, and its influence on photic entrainment is mediated by Shaggy, the kinase that phosphorylates TIM [11,79]. Octopamine mediates its effects on circadian rhythm by activation of different GTP-binding-protein (G protein)-coupled receptor types, which induce either cAMP production or Ca^2+^ release [80]. Dopamine receptor responsiveness is under circadian control and depends on the normal function of the *per* gene [81]. In addition, biogenic amines play an important role in the regulation of sleep, the physiological status of which is also adjusted by the circadian rhythm [82,83,84,85].

Finally, acetylcholine, glutamate and GABA have been implicated in circadian control in flies. Acetylcholinesterase is made in the sNPF^+^ LNds and in the fifth s-LNv, and the response of the sLNvs to acetylcholine suggests that the acetylcholine-positive LNds and the fifth s-LNv may communicate with the sLNvs with this neurotransmitter [49,86]. The sLNvs express the GluR and may respond to glutamate released by the DN1s and DN3s, thereby offering a way for these DNs to communicate with the sLNv’s [87,88,89]. Finally, GABA may act through GABAB receptors on the sLNvs, although it is not clear which cells provide the GABAergic input [90,91].

Different neuropeptides have been identified in a variety of neurons in different tissues, and they have different functions in the regulation of physiology and behavior, including circadian rhythm (for the details, see the review [65]). The neurons interact extensively via conventional neurotransmission and via neuropeptide signals to organize temporal information across the network and to mediate entrainment to environmental signals [88]. However, there is still much need to investigate neuropeptides’ functional loops and the relation between clock neurons and other neuronal components.

## 3. Circadian Rhythms, Neuropeptides and Neurodegenerative Disease in *Drosophila*

In humans, aging is associated with deficits in circadian rhythms and sleep and with a concomitant rise in neurodegenerative disease. The extent to which neurodegenerative disease is linked with circadian decline is an area of active investigation [92]. Are these pathologies independent consequences of aging, or does one cause the other (e.g., do circadian deficits contribute to neurodegeneration, or does neurodegeneration contribute to circadian deficits)? There are some indications that circadian or sleep deficits may precede obvious cognitive impairments associated with Alzheimer’s disease [93], consistent with a role for circadian or sleep pathology in the etiology of Alzheimer’s disease. On the other hand, expression of amyloid beta protein was already present in these patients, and expression of amyloid protein cleavage products in flies leads to circadian deficits [94,95].

The central circadian oscillations of gene products seems to persist in the suprachiasmatic nucleus (SCN) of the brain (viewed as the master circadian oscillator) in aging mammals, albeit with reduced amplitude [96,97,98]. Peripheral oscillators outside the brain can exhibit more extensive damped or reduced circadian regulation than the SCN [99], perhaps in part due to a reduced number of vasoactive intestinal peptide (VIP)-secreting cells (which is the mammalian equivalent of fly PDF), reduced number of electrically-active SCN cells and reduced phase coherence of the SCN [100,101,102].

The reduced phase coherence of the SCN and the damped oscillations of peripheral oscillators in mammals have parallels in analyses of links between circadian rhythms and neurodegeneration in flies. The oscillations in the central clock neurons of flies are not reduced in aging flies [103], while the oscillations of activity rhythms are reduced [103,104,105], most likely due to reduced peripheral oscillations of clock genes [103,105,106] or reduced output signaling from the central brain clocks (e.g., PDF) [107]. Moreover, old flies show reduced amounts of sleep [104], and flies expressing amyloid beta protein have circadian deficits that are not associated with deficits in circadian oscillations of the central brain oscillators, again suggesting defects in output pathways [94,95,108].

**Figure 3:**
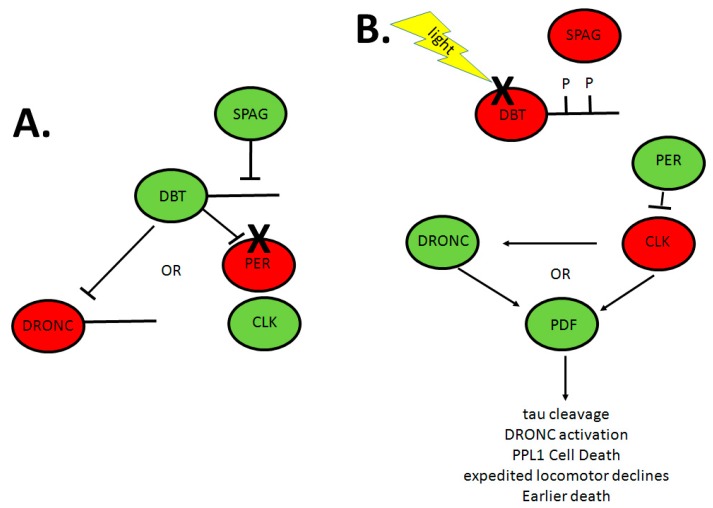
Hypothetical model for the PDF-dependent cell death/tauopathy pathway activated by reductions in DBT or CLK activity. Red proteins are in the inactive state and green proteins are in the active state. (**A**) In wild-type genotypes, SPAG associates with DBT to maintain it in the dephosphorylated state. DBT maintains the caspase DRONC in the inactive state and allows normal CLK activity via phosphorylation and degradation of PER; (**B**) With knock-down of SPAG, DBT autophosphorylates its C terminus and is degraded in response to light. This allows cleavage of DRONC to activate it and inactivation of CLK via accumulation of high levels of PER. A dominant negative DBT or inactive CLK mutation may likewise activate DRONC directly and/or via a PDF-dependent pathway. As a consequence, tau is cleaved, DRONC is activated, PPL1 neurons die, age-related locomotor declines are expedited and flies die sooner. Arrows represent positive action, and T-bars represent negative action. X indicates degradation. PDF, pigment dispersing factor; DBT, doubletime; CLK, clock; SPAG, spaghetti; PER, period; PPL1, posterior protocerebral lateral 1.

Our studies have shown a role for the circadian neuropeptide PDF in circadian modulation of caspase activation, which was correlated with enhanced tauopathy in a fly model for Alzheimer’s disease (Figure 3) [109]. We found that reductions in DBT activity (the fly ortholog of mammalian CKIδ/ε) in circadian cells lead to activation of DRONC (the fly ortholog of initiator caspase 9) in a light-dependent manner; in the middle of the day in an LD cycle or in the middle of the night after a 7-h light pulse. Reductions in DBT activity can be triggered by reductions in Spaghetti (SPAG), an HSP90 cochaperone, which associates with DBT to maintain its levels via antagonism of autophosphorylation of DBT’s C terminal domain and consequent proteasomal degradation [110], or by expression of a catalytically-inactive version of DBT (a dominant negative) in circadian cells. Activation of DRONC enhances the neurodegeneration produced by expression of human tau (htau) in the fly eye (the fly model for tauopathy) [109]. This enhancement correlates with reduced levels of full length htau protein, the cleavage of which is dependent on normal expression of DRONC. Caspase-dependent cleavage of htau has likewise been shown to facilitate production of tauopathy in human Alzheimer’s disease patients [111,112]. DBT activity apparently suppresses the activation of caspases, and reductions in its normal levels reveal a light-dependent pattern of elevated caspase activation in the middle of the day [109].

The strongly reduced levels of DBT in fly heads as a consequence of reduced SPAG levels only in circadian cells led us to investigating the cell autonomy of caspase activation. Intriguingly, activated caspase was detected broadly in the brain and especially in the optic lobes in areas innervated by PDF neurons, with general circadian cell expression of *spag* RNAi or the catalytically-inactive DBT using a *timeless* (*tim*)-GAL4 driver. In order to investigate the effects of PDF on the activation, we assessed caspase activation in response to more limited expression of catalytically-inactive DBT or *spag* RNAi under the control of a *pdf*-GAL4 driver, which is expressed only in the eight PDF^+^ cells in each brain hemisphere. This driver also produced expression of activated caspase in the optic lobes, and expression of activated caspase in the optic lobe was suppressed in a PDF receptor mutant background, thereby establishing that the activation was PDF dependent [109].

The results establish that PDF can signal activation of caspases in target cells (Figure 3). While the activation was only detected in circadian mutants of young flies, caspase activation was also detected in older wild-type flies, suggesting that circadian dysfunction arises in older flies mimicking that produced by mutations in younger flies [109]. Moreover, there is an extensive literature showing daily PDF-dependent changes in neuronal cell size and synaptic architecture in the optic lobes (reviewed in [113]). Since caspases have been implicated in synaptic pruning processes [114], it is possible that circadian dysfunction contributes to hyperactivation of this synaptic pruning pathway in circadian mutants or older wild-type flies. While activation of these caspases is transient in younger flies, flies with the *tim*GAL4 > *spag* RNAi genotype die sooner than wild-type flies, suggesting that there are negative consequences with time [109]. The mechanisms by which PDF signaling and reduced DBT activity activate the DRONC caspase are currently unknown. Caspase activation involves DRONC polypeptide cleavage, and this cleavage may be antagonized by phosphorylation, so it is possible that DRONC is directly phosphorylated by DBT to maintain it in the uncleaved inactive state.

On the other hand, it is possible that the circadian clock is linked to htau-induced neurodegeneration by suppressing other pathways that have been linked to neurodegeneration in flies and mammals, specifically oxidative damage pathways. In mammals, brain and muscle ARNT-like (BMAL)-mutant mice (carrying mutations in the mammalian equivalent of fly CYC) show enhanced oxidative damage to neurons [115,116,117]. Likewise, the *Drosophila per^o^* mutation leads to enhanced susceptibility to oxidative damage, accumulation of oxidized proteins and neuronal degeneration [118,119], and oxidative stress contributes to the break-down of the sleep-awake cycle in flies [104]. Finally, a recent RNA-seq analysis of old and young flies has shown that many stress-related genes exhibit robust daily oscillations of expression in old flies, but not in young flies (unless the young flies are subjected to oxidative stress) and that these oscillations require the *Clk* gene [120]. Since a mutation in *Clk* also leads to activation of DRONC in a light-dependent manner during the middle of the day, it is possible that the effects we have detected are produced by downstream transcriptional consequences (e.g., high levels of PER and low CLK activity) of DBT reductions ([109] and Figure 3), as they are with the BMAL-mutant mice. On the other hand, expression of DBT alone (without PER or CLK) in *Drosophila* S2 cells antagonizes UV-induced cell death in a manner that requires autophosphorylation of its C terminal domain [110], and knock-down of DBT alone in S2 cells activates DRONC and cleaves co-expressed tau [109].

Other evidence for a PDF-dependent role in circadian-relevant neurodegeneration has come from analysis of locomotor activity declines produced during aging [121]. These declines are more rapid in the *Clk^AR^* mutant and are due to loss of CLK function in the PDF^+^ neurons. The deficits were linked to losses in dopaminergic neurons in the PPL1 cluster, and these losses require the presence of the PDF receptor and functional apoptosis pathway (Figure 3), again suggesting the regulation of apoptosis via the PDF receptor. In this case, the activation of the cell death pathway was shown to produce the loss of specific neurons that drive locomotor activity and to be independent of the circadian clock phenotype, lifespan deficits and reactive oxygen species accumulation that are also associated with *Clk^AR^*.

Other circadian neuropeptides may also contribute to circadian regulation of neurodegeneration. Insulin/insulin-like growth factor-dependent signaling has been shown to regulate lifespan and health span in a number of organisms, including *Drosophila*. This pathway interacts with circadian pathways via AKT/TOR-S6 signaling, which affects the circadian period in *Drosophila* [122]. Decreases in insulin and TOR signaling can reduce sleep deficits related to aging in flies [123], thereby providing a potential link between this pathway and circadian/sleep-deprived effects on neurodegeneration. Finally, expression of *dfmr1* (the *Drosophila* ortholog of Fragile X mental retardation protein) in the insulin-secreting cells of the fly brain is sufficient to restore circadian and memory deficits that are produced in the fly model for the Fragile X syndrome neurodevelopmental disorder [58]. Dilp2 is elevated in the fly *fmr1* mutant, and levels are reduced by rescue with wild-type FMR1 in the insulin-secreting cells. Mutations that reduce insulin signaling ameliorate the effects of *fmr1* mutations on circadian and memory pathways in flies [58]. All of these results are consistent with a role for insulin-signaling in the circadian and cognitive effects of this neurodevelopmental disorder.

Finally, other proteins involved in neurodegenerative diseases have been linked to the circadian mechanism in *Drosophila*, although the relevance of their linkage to any interaction between the circadian mechanism and neurodegenerative disease is not yet clear. Atxn-2, an RNA-associated protein involved in neurodegenerative disease, is required for normal PER translation [31,124]. Another example is pantothenate kinase; reduction of this kinase in circadian neurons affects circadian rhythms and produces several aspects of the neurodegenerative disease to which it is related in humans [125].

## 4. Perspectives

With the fast pace of society and the increasing amount of night-shift work, circadian rhythm and sleep disorders as a health hazard are now taken seriously. Like *Drosophila*, mammals also produce the classical circadian cycles. A heterodimer of CLOCK and BMAL1 promotes the transcription of *per* and *cry*. Two mammalian CRY proteins can bind to three different PER proteins and repress CLOCK/BMAL1 activity [126,127]. The studies on circadian rhythms in *Drosophila* provide much valuable information on the mechanism of internal clock interactions with environmental clues and physiological processes, and most of them are applicable to mammals.

Like the fly clock, the mammalian organismal clock contains multiple cellular and tissue oscillators that must be maintained with the appropriate relative phases. The role of neuropeptides in the maintenance of this synchrony is clearly relevant to human health, as the above discussion of human and fly neurodegeneration has implied. It is clear that studies employing the powerful genetic techniques of *Drosophila* have already helped to elucidate the general principles of circadian output pathways, both for normal circadian function and for circadian dysfunction produced by aging, and that they will continue to do so in the future.
